# Electroacupuncture for abdominal pain relief in patients with acute pancreatitis: study protocol for a randomized controlled trial

**DOI:** 10.1186/s13063-018-2644-1

**Published:** 2018-05-16

**Authors:** Dong Kee Jang, Chan Yung Jung, Kyung Ho Kim, Jun Kyu Lee

**Affiliations:** 1 Department of Internal Medicine, Dongguk University College of Medicine, Dongguk University Ilsan Hospital, 27 Dongguk-ro, Ilsandong-gu, Goyang, 10326 Korea; 2 Institute of Oriental Medicine, College of Korean Medicine, Dongguk University, Gyeongju, Republic of Korea; 3 Department of Acupuncture and Moxibustion, Dongguk University College of Korean Medicine, Dongguk University Ilsan Oriental Hospital, Goyang, Republic of Korea

**Keywords:** Acute pancreatitis, Electroacupuncture, Pain management, Abdominal pain

## Abstract

**Background:**

Previous studies have shown that electroacupuncture (EA) reduces the severity of acute pancreatitis. However, the effect of EA for pain relief in patients with acute pancreatitis has not been evaluated yet. The purpose of this study was to prove the efficacy of EA for pain relief in patients with acute pancreatitis compared with conventional treatment.

**Methods:**

This study is a randomized, controlled, three-arm, parallel-group, multi-center trial. Patients diagnosed with acute pancreatitis are enrolled and randomly assigned to EA 1, EA 2, or a control group in a 1:1:1 ratio. All the enrolled patients basically receive the conventional standard-of-care therapy for acute pancreatitis. Local EA is given in group EA 1, while local with additional distal EA is given in group EA 2. Local EA includes two acupoints, *Zhong Wan* (CV12) and *Shang Wan* (CV13), located in the abdomen, while distal EA includes 12 peripheral acupoints, *Zhong Wan* (CV12), *Shang Wan* (CV13), *He Gu* (LI4), *Nei Guan* (PC6), *San Yin Jiao* (SP6), *Xuan Zhong* (GB39), *Zu San Li* (ST36), and *Shang Ju Xu* (ST37). The patients randomized to the EA 1 and EA 2 groups undergo one session of EA daily from day 1 until day 4, or until pain resolves. The primary endpoint is the Visual Analog Scale (VAS) change for pain on day 5. Secondary endpoints include daily VAS, requirement of analgesics, changes of inflammatory markers, time to pain disappearance, and hospital days.

**Discussion:**

The results of this trial are expected to prove the efficacy of EA for pain relief in patients with acute pancreatitis. Based upon the results, EA would be applied to a variety of clinical practices for reducing pain.

**Trial registration:**

This trial is registered at ClinicalTrials.gov, ID: NCT03173222. Registered on 1 August 2017.

**Electronic supplementary material:**

The online version of this article (10.1186/s13063-018-2644-1) contains supplementary material, which is available to authorized users.

## Background

The prevalence of acute pancreatitis (AP) is increasing due to cholelithiasis and increased alcohol consumption [1]. While the overall mortality in all hospitalized patients with AP is approximately 10%, the mortality in the subset with severe AP may be as high as 30% [2]. AP causes significant abdominal pain, which can adversely affect patients’ quality of life. Most patients have associated nausea and vomiting [3]. Pain control for such patients is the mainstay of AP management.

Opioids are safe and effective for pain control in patients with AP. Compared with other analgesic options, opioids may decrease the need for supplementary analgesia [4]. However, frequent administration of opioid analgesics may result in opioid dependency [5, 6]. In the United States, there has been a tripling in the rate of opioid-related overdose deaths from 2000 to 2014, with over 28,000 deaths in 2014 [7]. This epidemic creates a dilemma for physicians who seek to provide adequate pain relief while minimizing risks of abuse and dependency [8]. Unfortunately, any innovative treatment for better analgesia than opioids has not been developed in medical science so far. Therefore, the development of a novel treatment for pain relief without triggering dependency is urgent.

Acupuncture originated in China approximately 2,000 years ago and is one of the oldest medical procedures in the world. Acupuncture continued to be developed and codified in texts over the subsequent centuries and gradually became one of the standard therapies used in China. Later, acupuncture was introduced to other regions including Asia, Europe, and the United States [9]. The most thoroughly studied application of acupuncture is for pain relief [10]. The proposed mechanism of action was that acupuncture stimulation is associated with neurotransmitter effects such as endorphin release [11]. In this sense, acupuncture within oriental medicine could be an appropriate alternative for opioid analgesics, since it has been known as an effective treatment for pain relief without dependency. Electroacupuncture (EA) is a form of acupuncture where a small electric current is passed between pairs of acupuncture needles. Previous studies have suggested that EA blocks pain by activating a variety of bioactive chemicals through peripheral, spinal, and supraspinal mechanisms [12]. A recent clinical trial showed that EA reduced the duration of postoperative ileus, time to ambulation, and postoperative analgesic requirement, compared with no or sham acupuncture, after laparoscopic surgery for colorectal cancer [13]. Another study indicated that EA may reduce the severity of AP by inducing anti-inflammatory effects [14]. However, to the best of our knowledge, the effect of EA for pain relief in patients with AP has not been evaluated yet. Therefore, we planned to prove the efficacy of EA for pain relief in patients with AP compared with conventional treatment.

## Methods

### Study design and ethical considerations

This study is a randomized, controlled, three-arm, parallel-group, prospective, multi-center trial conducted for the purpose of comparing and analyzing the efficacy of pain relief between conventional treatment and EA + conventional treatment in patients with abdominal pain who are diagnosed with AP. It is designed as a three-arm, parallel-group, superiority trial. All participating hospitals are teaching hospitals, which have both College of Medicine and College of Korean Medicine recognition. This study was approved by the Institutional Ethics Review Board (IRB) of Dongguk University Ilsan Hospital (IRB No. 2017-50; Dongguk University Ilsan Oriental Hospital, 2017-06). Based on the Helsinki Declaration, the health and human rights of subjects will be prioritized and voluntary written consent will be obtained from all subjects before entering clinical studies. We will obtain all informed consents after 30 min of explanation. Records identifying the subject’s identity will be kept confidential.

### Inclusion criteria

Inpatients who meet the following requirements are eligible for enrollment: (1) diagnosed with AP; the definition of AP is based on the fulfillment of two out of three of the following criteria: clinical (upper abdominal pain), laboratory (serum amylase or lipase > 3× upper limit of normal) and/or imaging (computed tomography, magnetic resonance imaging, ultrasonography) criteria [2]; (2) aged ≥ 20 years; (3) understand the nature and the risks involved in the study and are able to communicate with the investigators; and (4) signed the written informed consent by listening to the explanation of the purpose, methods, and effects of this study.

### Exclusion criteria

Participants with any of the following conditions are excluded: (1) severe AP with multi-organ failure; (2) history or intolerance to EA; (3) pregnant or breast-feeding women; (4) informed consent could not be obtained; and (5) considered to be difficult to perform this clinical trial when judged by the principal investigator.

### Randomization

Patients will be randomized in a 1:1:1 fashion to receive EA1, EA2, and the control group. Block randomization is performed using Random Allocation Software version 1.0.0. A person who is not involved this study creates random numbers before the first subject enrolls after IRB approval. Then, the random numbers are kept in sealed envelopes in the order of their creation. An independent hospital staff member will prepare the sealed envelopes to ensure concealment of the allocation sequence. The numbers are handed over to assistants (independent researchers). After randomization, the treatment method is not blinded.

### Standard-of-care therapy

All the enrolled patients basically receive the conventional standard-of-care (SOC) therapy for AP. For the conventional therapy, first, non-steroidal anti-inflammatory drugs are administered; afterwards, if inadequately controlled, low-potency narcotic analgesics, such as codeine, and then high-potency narcotic analgesics, such as morphine, will be given sequentially as required [15].

### Interventions and electroacupuncture procedures

**Fig. 1:**
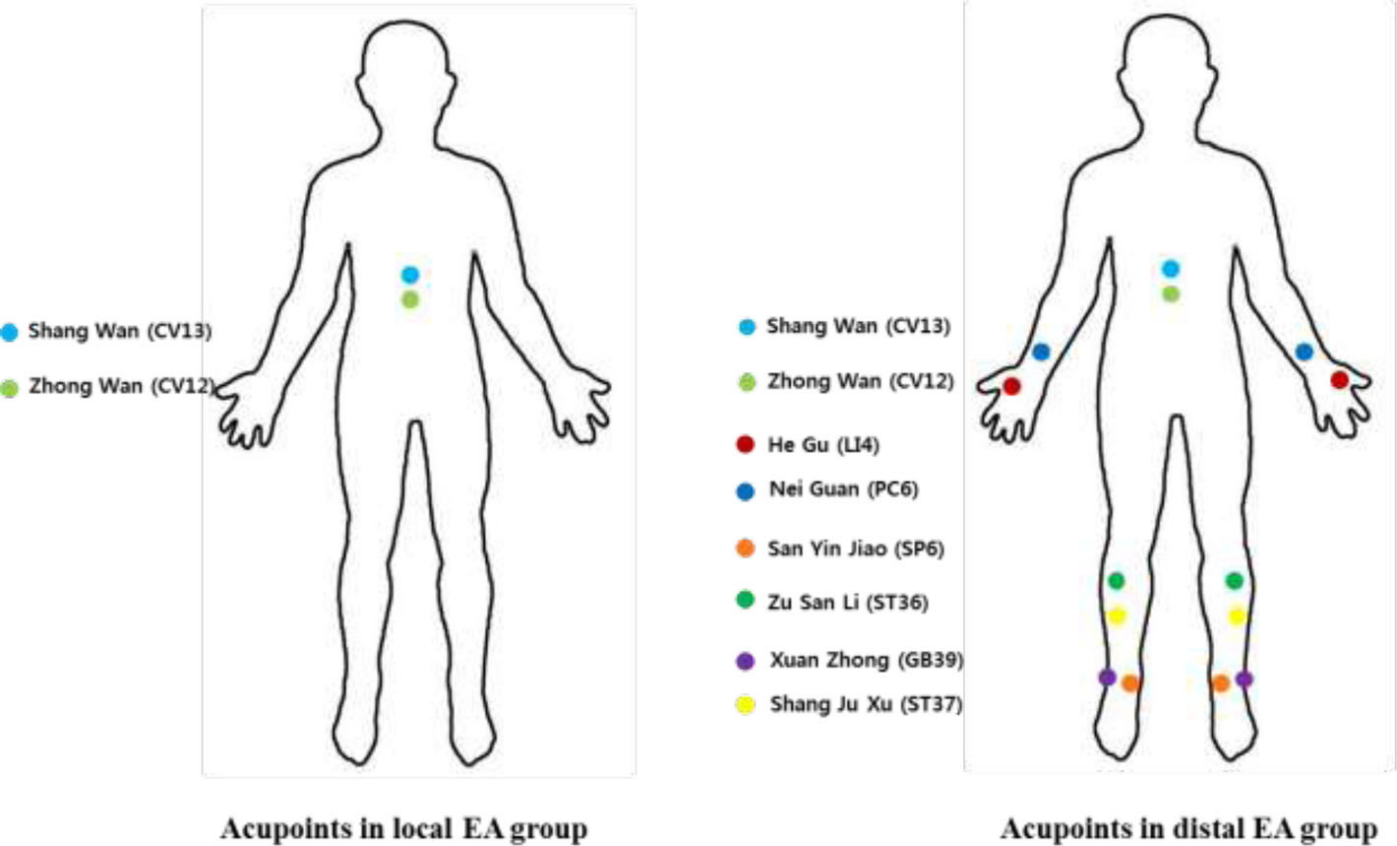
Locations of acupoints in electroacupuncture groups

Local EA is given in group EA 1, while local with distal EA is given in group EA 2, additionally. The EA 1 group receives the same SOC therapy in combination with EA therapy at a total of two acupoints including *Zhong Wan* (CV12) and *Shang Wan* (CV13). The EA 2 group also receives the same SOC therapy in combination with EA therapy at a total of 14 acupoints including *Zhong Wan* (CV12), *Shang Wan* (CV13), *He Gu* (LI4), *Nei Guan* (PC6), *San Yin Jiao* (SP6), *Xuan Zhong* (GB39), *Zu San Li* (ST36), and *Shang Ju Xu* (ST37) (Fig. 1) [14, 16, 17]. Electric stimulation is applied with mixed frequencies (30 and 3 Hz). Each EA session lasts 15 min (STN-330 electronic stimulator, Stra Tek Co., Ltd., Gyeonggi-do, Korea). Both EA groups receive auricular acupuncture therapy at a total of five auricular acupoints, including Sympathetic, *Shen Men*, Abdomen, Pancreas gall, and Spleen, alternately at the right or left side, whenever EA is given. Both EA groups undergo one session of EA daily from day 1 until day 4, or until pain resolves.

### Outcomes

The primary endpoint is the change in Visual Analog Scale (VAS, 0–100) for pain on day 5 compared to pretreatment. Secondary endpoints include daily VAS (days 1–4), requirement of analgesics, changes of inflammatory marker (C-reactive protein), time to pain disappearance and hospital days. Daily VAS is the average of VAS measured three times a day. The requirement of analgesics includes both the amount and frequency of analgesics administered daily.

### Planned number of subjects and period

**Fig. 2:**
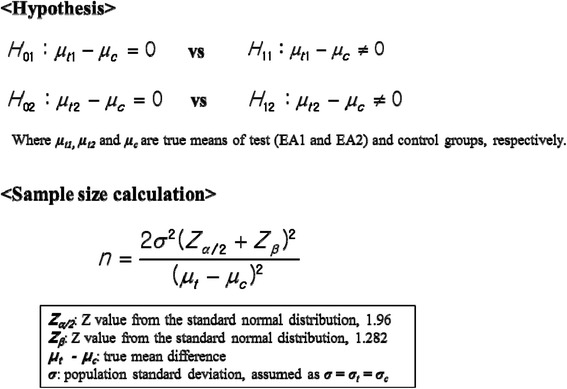
Sample size calculation

**Fig. 3:**
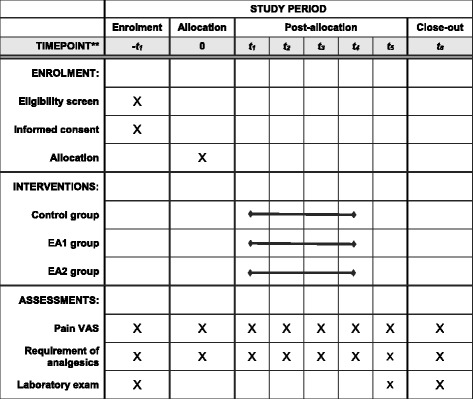
Schedule of enrollment, interventions, and assessments*

A total of 99 patients will be enrolled for 2 years. Sample size calculation was performed for achieving a 90% power at the 5% level of significance.. The sample size was calculated as in Fig. 2. The population standard deviation (*σ*) was determined as the largest value according to a previous study [18]. Therefore, the standard deviations are 19 for the test group and 25 for the control group. The expected minimum clinical difference of 20 has been defined; this is the difference between the test group and the control group. This is achieved by enrolling 25 evaluable patients in each treatment group. To allow for a possible 20% dropout rate, 33 patients will be randomised to each group. The enrolled patients will be followed up for 1 month to monitor any delayed adverse events (see Fig. 3 for the schedule of enrollment, interventions, and assessments..

### Adverse events

Adverse events refer to undesirable and unintended signs, symptoms, or illnesses that occur after the procedure of a clinical research process. Adverse events related to acupuncture treatment are harmful and unintended reactions that cannot be excluded from the causal relationship with acupuncture used in this study. The types of adverse events include subcutaneous hemorrhage (2.9%), autonomic nervous system abnormalities (1%) such as hypotension and excessive sweating, and gastrointestinal symptoms (< 1%) such as nausea and vomiting. Significant adverse events include those that cause death or threaten life, require an extension of hospitalization or length of stay, cause disability, impairment of function, or congenital malformations. Even if not in the circumstances listed above, a medical condition that is likely to have a significant impact on the patient’s well-being and health condition is regarded as a significant adverse event (SAE) by the medical judgment of investigators and relevant experts. All adverse events will be recorded in the Case Report Form, and SAEs will be reported to the IRB as well as on the ClinicalTrials.gov database, and managed appropriately. The IRB will decide whether to stop or continue this study when SAEs occur.

### Data management

Double checks for data values will be performed by a trained research assistant after completion of final data collection. A data and safety monitoring committee is not required since the acupuncture study has minimal risks. Periodic data monitoring and audit for the important aspects of the trial will be conducted by two independent, trained investigators. The final data will be assessed by the first author and independent statistician. An interim analysis is not planned. The information of patients discharged before day 5 will be collected from telephone or outpatient clinic at day 5, which will be included in the analysis. For those who died during the study, only the data before death are included in the analysis.

### Statistical analysis

The statistical analysis will be based on the intention-to-treat (ITT) population. Missing data imputation will be used in order to evaluate the robustness of the primary endpoint analyses. The primary imputation method will be LOCF (last observation carried forward). The statistical analysis will be conducted for both three (EA 1: EA 2: control) and two (EA: control) groups. The null hypothesis is that there is no difference in the changes of variables between the two or three groups before and after treatment. The three groups will be compared by one-way analysis of variance (ANOVA) or the Kruskal-Wallis test for continuous variables, and chi-squared or Fisher’s exact test for categorical variables. Analysis of covariance will be performed with covariates that may affect VAS level. Post hoc analysis will be done if there is a significant difference between the three groups using the Bonferroni correction. The two (EA: control) groups will be compared by means of Student’s *t* test or Wilcoxon’s rank sum test for continuous variables, and chi-squared or Fisher’s exact test for categorical variables. A two-sided *P* value of less than 0.05 will be considered to indicate statistical significance.

## Discussion

AP is one of the most common diseases of the gastrointestinal tract, leading to serious emotional, physical, and financial human burden [19]. Patients with AP typically present with sudden epigastric or left upper quadrant pain. Severe abdominal pain is the hallmark symptom of AP, and sometimes radiates to the back, chest, or flanks [3]. Interestingly, the severity of pain may correlate with the severity of AP [20]. Therefore, aggressive control of pain can help to improve the quality of life and reduce the severity of pancreatitis in patients with AP, regardless of etiology. An effective treatment of pain in AP ranges from the administration of simple analgesic drugs, such as non-steroidal anti-inflammatory drugs, up to the administrations of potent opioid drugs according to the severity [4, 21]. However, the adequate treatment of pain is much more complex in clinical circumstances. While the World Health Organization (WHO) regimen is usually applied to the pain management [15], some interventional strategies should be considered in patients with severe AP-related complications. One reason for the challenge behind pain management is the high complexity of AP itself. Patients with necrotizing AP often suffer from severe pain attacks, associated pleural effusion , ascites, and even multiple organ failure. Despite the importance of controlling abdominal pain, most guidelines do not provide precise guidelines for abdominal pain relief [1–3].

Until the 1990s, opioid analgesics were used only for the treatment of moderate to severe acute pain and cancer pain, but recently, there has been an increase in long-term use for non-cancer pain such as in pancreatitis. Theoretically, because there is no ceiling effect in the use of opioid analgesics, they can be an attractive choice for the pain without improvement by non-analgesic agents [22, 23]. However, 80% of patients using opioid analgesics experience at least one side effect. The most common side effect is constipation. Vomiting, sedation, and drowsiness are also relatively common, and respiratory failure can occur if the initial dose is excessive or increases rapidly. In addition, long-term use may also cause serious side effects such as hyperalgesia, neuroendocrine dysfunction, and immunosuppression. Above all, the continued use of opioid analgesics can cause patients to become more physically and psychologically addicted to the drug over time [24]. Therefore, there is an urgent need to develop safe and effective methods to replace the use of opioid analgesics in the management of pain in patients with AP.

Acupuncture is already proven to be effective for a variety of pain types including abdominal pain [10], and there is no concern about dependency, so it can be a good alternative to opioid analgesics for the treatment of pancreatitis pain. This trial started from this idea at first, and was planned primarily based upon the previous study on patients with colorectal cancer surgery [13]. The study suggested that EA can reduce postoperative pain as well as the duration of postoperative ileus. The mean VAS of EA group on day 3 was 2.1 ± 1.2, which was significantly lower than that of sham group (3.2 ± 1.8, *P* < 0.001). Another previous study also has shown that EA could reduce the postoperative analgesic requirement and associated side effects in patients with abdominal surgery [25]. The analgesic effect induced by EA might be mediated by κ-opioid receptors through the release of dynorphin [26].

In this study, we aimed to demonstrate the hypothesis that EA would be more effective in pain control than SOC therapy in patients with AP. We set two different treatment groups, local and distal EA, to compare the effects according to acupoints. The two acupoints (CV12, CV13) in the local EA group are located in the abdomen, which might affect abdominal pain due to the location. Therefore, we have introduced the other arm, the distal EA group, which includes distal acupoints located in the arms and legs. The treatment in the distal EA group is expected to mask the location effect in the abdomen. Furthermore, the comparison between the two EA groups will provide valuable information on the effect of distal acupuncture in patients with abdominal pain. To the best of our knowledge, this trial is the first randomized controlled study to introduce EA, which is the representative method of oriental medicine, in the treatment of AP. Therefore, irrespective of the result, this study should give a critical result on the effect of EA. If the efficacy of EA is proven to be significant, changes in guidelines for the management of AP are expected in the future (Additional file 1).

## Trial status

This trial is currently recruiting patients, and expected to be finished on 29 February 2020.

## Additional file

Additional file 1: SPIRIT 2013 Checklist: Recommended items to address in a clinical trial protocol and related documents. (DOCX 59 kb)

## Abbreviations

AP: Acute pancreatitis
EA: Electroacupuncture
IRB: Institutional Ethics Review Board
SOC: Standard-of-care
VAS: Visual Analog Scale
